# Cryoglobulinemia Associated With Multiple Myeloma in a Dog Presenting With Epistaxis and Skin Lesions

**DOI:** 10.1002/vms3.70084

**Published:** 2024-10-20

**Authors:** Myrto Spyropoulou, Ivan Montanes‐Sancho, Adam G. Gow, Suzanne Bussey

**Affiliations:** ^1^ Easter Bush Pathology The Royal (Dick) School of Veterinary Studies and The Roslin Institute, University of Edinburgh Edinburgh UK; ^2^ Hospital for Small Animals The Royal (Dick) School of Veterinary Studies and The Roslin Institute, University of Edinburgh Edinburgh UK

**Keywords:** blood, canine, haematology, lymphoproliferative, monoclonal, neoplasia

## Abstract

A 10‐year‐old female neutered Labrador Retriever presented with epistaxis, discoloration and crusting of the nose and a necrotic lesion on the lip. Bloodwork revealed pancytopenia, azotemia, hypoalbuminemia and hyperglobulinemia. Aggregates of amorphous basophilic material were seen in a room‐temperature blood smear which were not present in the sample after warming to 37°C, and grossly a cryoprecipitate was noted in the patient's serum at 4°C. This was interpreted as cryoglobulin. Computed tomography showed multiple heterogeneous lesions in the spleen. Cytology of the splenic lesions revealed marked plasma cell infiltration, consistent with neoplasia. Bone marrow aspiration revealed an increased proportion of plasma cells (approximately 38% of the total cells). Serum protein electrophoresis showed a monoclonal spike in the gamma globulin region. A diagnosis of multiple myeloma associated with cryoglobulinemia was made. The patient received palliative care with prednisolone while the owner was considering chemotherapy. However, she rapidly deteriorated and was euthanized. The combination of cryoglobulin precipitation and hyperviscosity syndrome was considered responsible for the patient's original symptoms. Cryoglobulinemia is an extremely rare phenomenon that is often associated with lymphoproliferative disorders. This report describes its association with multiple myeloma in a dog presenting with atypical initial signs.

## Graphical abstract

Blood smears made from the chilled EDTA were dominated by multifocal aggregates of light basophilic material. These aggregates were absent from the blood smears made by the same EDTA after the tube had been warmed. This phenomenon was consistent with cryoglobulin precipitation and has been previously described in dogs with lymphoproliferative diseases. Bone marrow cytology from this patient showed high numbers of plasma cells, and serum protein electrophoresis revealed the presence of monoclonal gammopathy. The patient was diagnosed with multiple myeloma, associated with cryoglobulinemia.

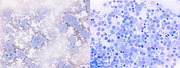

## 1 Case Presentation

**FIGURE 1:**
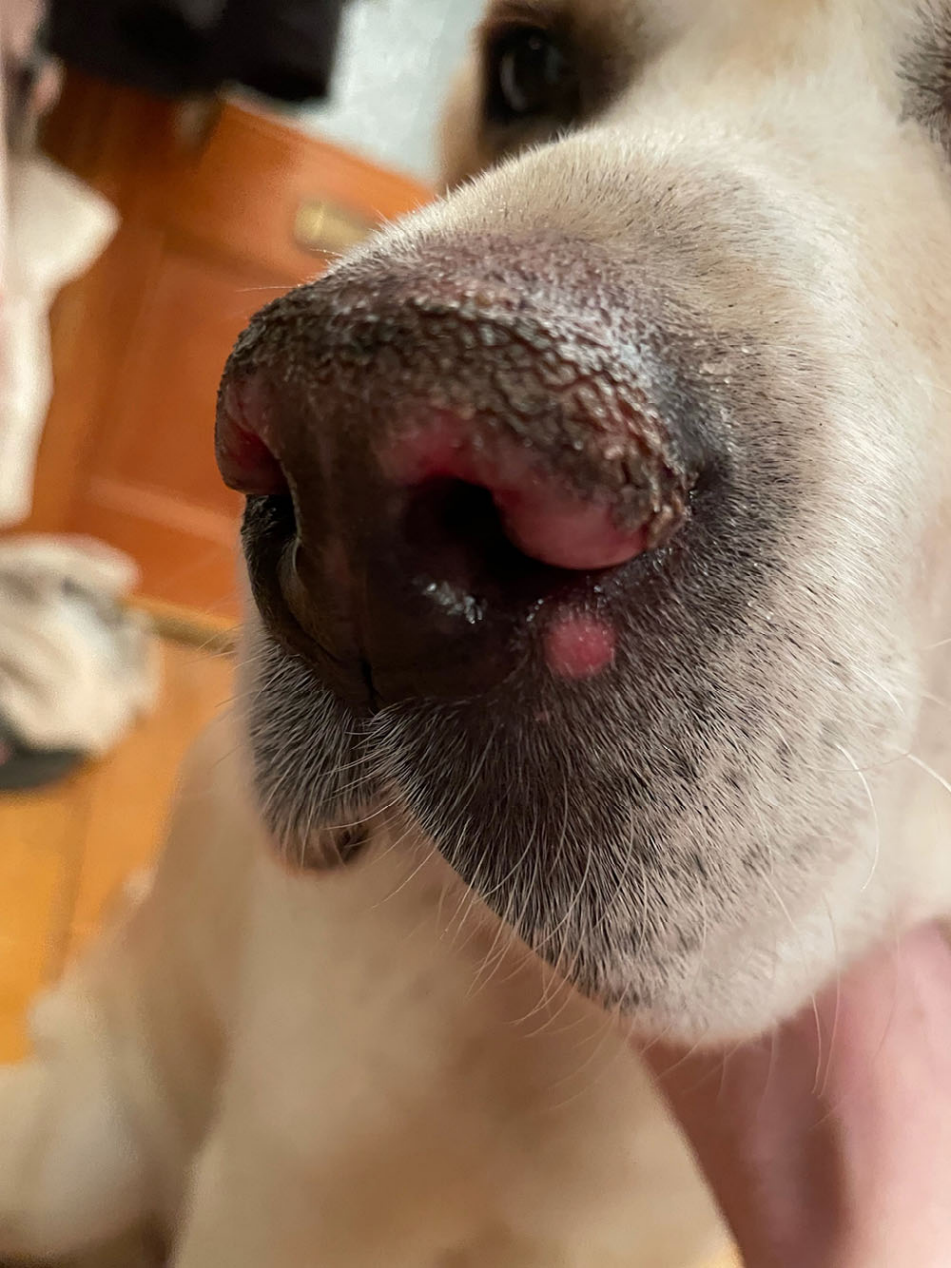
Photograph of the skin and nasal planum lesions of a Labrador Retriever dog, showing diffuse symmetrical hyperkeratosis and crusting of the nasal planum with fissure formation. There is additionally diffuse ulceration/erosion on the alar folds and a circular focal erosion on the haired skin ventral to the left naris.

A 10‐year‐old female neutered Labrador Retriever presented to a primary care veterinary practice, with a history of left‐sided epistaxis and discolouration of the nose of approximately 10 days duration. No response to symptomatic treatment with antibiotic (clindamycin, Zodon 10 mg/kg PO BID) and non‐steroidal anti‐inflammatory (firocoxib, Previcox 3.8 mg/kg PO SID) medications was noted, with worsening of the epistaxis, which became bilateral. Additionally, marked crusting of the nasal planum and an ulcerated and necrotic lip lesion developed. After 3 weeks, the patient was referred to the internal medicine service at the Hospital for Small Animals at The University of Edinburgh for further investigations. At presentation, the patient had also developed a mild intermittent cough, and epistaxis episodes were ongoing. The nose had depigmentation of the dorsal alar folds with some ulceration, hyperkeratosis and fissuring of the nasal planum (Figure 1). She was still receiving the symptomatic treatment prescribed by the referring veterinarians. She was otherwise bright, alert and responsive, with no other abnormal findings on physical examination. Initial clinical differential diagnoses based mainly on the chronic bilateral nasal discharge, nasal discoloration and crusty nose included aspergillosis, immune‐mediated disease (e.g., discoid lupus erythematosus), infectious disease (e.g., leishmaniosis) or nasal neoplasia.

Routine haematology and biochemistry were performed at Easter Bush Pathology at The University of Edinburgh using the Siemens Advia 2120 and Beckman Coulter AU480, respectively. Analysis was carried out within 2 h of sampling, using an EDTA and serum tube, respectively, at room temperature. Initial bloodwork (Table 1) revealed a moderate normocytic, normochromic, non‐regenerative anaemia, mild neutropenia and thrombocytopenia no evidence of platelet clumping was seen on blood smear examination). On biochemistry (Table 2), the patient was noted to be moderately azotemic. Marked hypoalbuminemia and mild hyperglobulinemia were also noted, with the total protein within reference interval (RI). The rest of the biochemistry profile and coagulation parameters were unremarkable.

**TABLE 1:** Hemogram results (Advia 2120).

Test	Result	Reference interval	Units
HCT	**0.25**	0.39–0.55	L/L
RBC	**3.82**	5.50–8.50	× 10^12^/L
HGB	**8.8**	12.0–18.0	g/dL
MCV	65.7	60.0–77.0	fL
MCHC	35.0	32.0–36.0	g/dL
RDW	15.6	N/A	%
Reticulocytes (absolute count)	23.9	0.0–60.0	× 10^9^/L
WBC	**5.36**	6.00–15.00	× 10^9^/L
Segmented neutrophils	**2.95**	3.60–12.00	× 10^9^/L
Non‐segmented neutrophils	0.00	0.00–1.00	× 10^9^/L
Lymphocytes	1.77	0.70–4.80	× 10^9^/L
Monocytes	0.32	0.00–1.50	× 10^9^/L
Eosinophils	0.32	0.00–1.00	× 10^9^/L
Basophils	0.00	0.00–0.20	× 10^9^/L
Platelets	**178**	200–500	× 10^9^/L

Values outside the reference interval are written in **bold**.

**TABLE 2:** Selected biochemistry (Beckman Coulter AU480) and coagulation results (Stago STart Max).

Test	Result	Reference interval	Units
Total protein	59.9	58–73	g/L
Albumin	**15.6**	26–35	g/L
Globulin	**44.3**	18.0–37.0	g/L
Urea	23.9*	1.7–7.4	mmol/L
Creatinine	230.0*	22.0–115.0	µmol/L
Total calcium	2.4	2.3–3.0	mmol/L
Ionized calcium	1.36	1.15–1.5	mmol/L
PT	7.8	5.0–12.0	Seconds
APTT	11.6	10.0–20.0	Seconds
Fibrinogen	2.8	2.0–4.0	g/L

Values outside the reference interval are written in **bold**.

**FIGURE 2:**
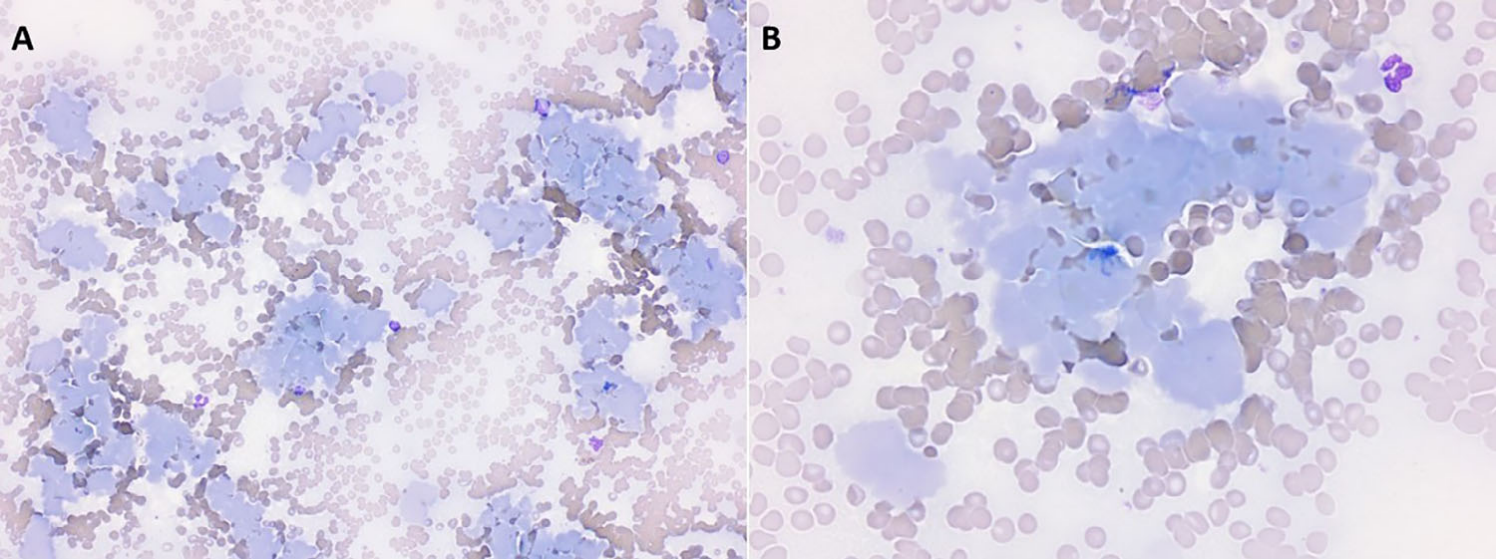
Photomicrograph of aggregates of amorphous basophilic material on the blood smear from a dog, made from a room temperature EDTA blood sample. Wright‐Giemsa, ×20 objective (A) and ×50 objective (B).

**FIGURE 3:**
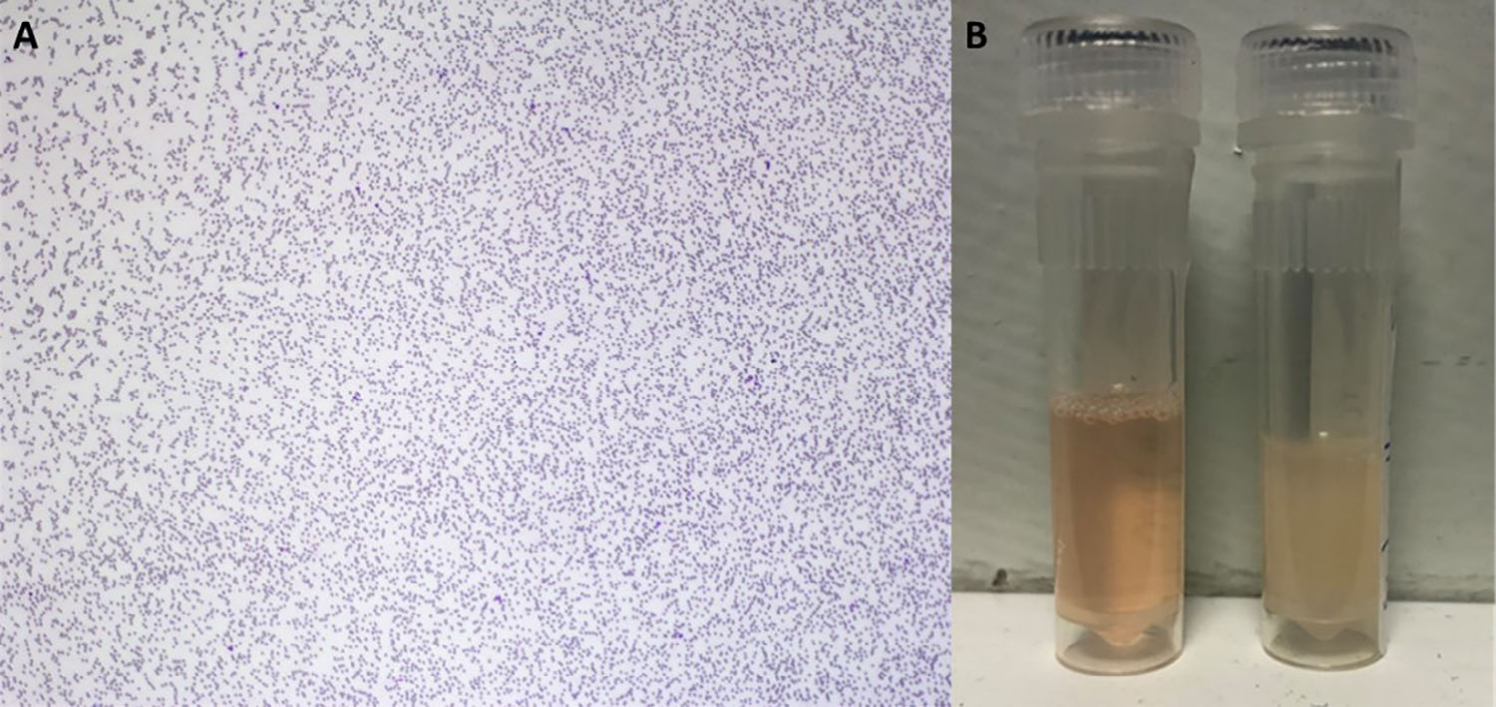
Photomicrograph of the blood smear from a dog, made from a warmed EDTA blood sample. Wright‐Giemsa, ×4 objective (A). Photograph of the warmed and cooled serum sample; note the cloudiness of the refrigerated serum sample, while the warmed serum is clear (B).

Multiple blood smears were made from the room temperature EDTA sample and submitted for examination by a resident and board‐certified clinical pathologist. The slides were dominated by the presence of abundant, large, multifocal aggregates of smooth, amorphous, lightly basophilic to amphophilic material (Figure 2A,B). Rare reactive lymphocytes were present. No other morphologic abnormalities were seen. The large amounts of amorphous material were suspected to be proteinaceous in nature, with immunoglobulin and fibrinogen considered possible. The EDTA sample was warmed to 37°C and new smears were made; the previously described material was absent on smear examination (Figure 3A). The serum was divided into two aliquots: One was placed in the fridge at 4°C, and the other was warmed to 37°C. After several hours (approximately 4–8 h), the cooled sample appeared opaque and contained a gelatinous material (cryoprecipitate), whereas the warmed sample was clear (Figure 3B). The presence of cryoprecipitate in the serum and blood smear, which dispersed after warming of the samples, was attributed to cryoglobulin.

Biochemistry was repeated with a cooled and a warmed sample, using the IDEXX Catalyst Dx chemistry analyser. A significant discrepancy was noted, with a marked hyperglobulinemia, and hyperproteinemia observed in the sample warmed to 37°C, compared to the cooled sample (Table 3). This discrepancy was attributed to the precipitation of immunoglobulins in the room temperature sample, causing a falsely low value.

**TABLE 3:** Comparison between selected serum biochemistry on the sample at room temperature (Beckman Coulter AU480) and the cooled and warmed samples (Catalyst Dx).

Test	Room temperature (Beckman Coulter AU480)	Cooled sample (Catalyst Dx)	Warmed sample (Catalyst Dx)
Total protein (g/L)	59.9 (58–73)	65 (52–82)	**90** (52–82)
Albumin (g/L)	**15.6** (26–35)	19 (22–39)	**20** (22–39)
Globulin (g/L)	**44.3** (18–37)	**46** (25–45)	**70** (25–45)
Alb/Glob	0.35	0.41	0.3

*Note*: Reference intervals for each analyser are shown in parenthesis next to the obtained value.

Values outside the reference interval are written in **bold**.

**FIGURE 4:**
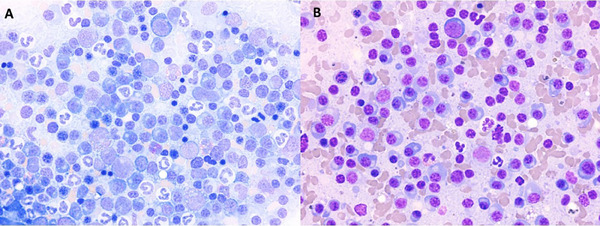
Photograph of the cytologic appearance of the bone marrow (A) and spleen (B) from a dog with cryoglobulinemia. May‐Grunwald Giemsa, ×50 objective. High proportions of mainly well‐differentiated plasma cells are present, consistent with multiple myeloma.

Further clinical examination of the patient under sedation revealed additional mucocutaneous areas of ulceration (3 cm linear ulcer on right lip margin and focal area of erythema and ulceration in the vulva). An abdominal ultrasound showed several rounded, well‐defined, hypoechoic and fairly heterogeneous lesions within the spleen, varying in size, with the largest (approximately 1.5 cm in diameter) located at the level of the body. Computed tomography of the head, neck and thorax identified bilateral, mild, non‐destructive rhinitis. No bone lesions were seen. Fine needle aspirates of the splenic lesions revealed marked expansion of plasma cells, comprising mostly mature and occasional immature forms, alongside a mixed background lymphoid population (Figure 4B). These findings were consistent with plasma cell neoplasia.

Given the findings for the spleen, and considering also the pancytopenia, bone marrow aspiration and biopsy were performed and submitted for examination (Figure 4A). The bone marrow was hypercellular with a moderate plasma cell expansion (approximately 38% of the total nucleated cells, with the cellular trails containing higher proportions of plasma cells), comprising mostly mature but also immature forms, as well as rare binucleated plasma cells. The erythroid, myeloid and megakaryocytic cell lines appeared normocellular. Given the presence of multiple peripheral cytopenias, in the face of the neoplastic infiltration of the marrow, a degree of myelophthisis was suspected. Rare osteoclasts were also present, suggesting bone remodelling.

**FIGURE 5:**
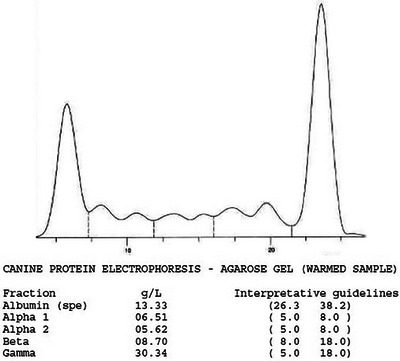
Serum protein electrophoresis from a dog with cryoglobulinemia. The spike in the region of gamma globulins, narrower and higher than the albumin, is consistent with a monoclonal gammopathy.

Serum protein electrophoresis (SPE) performed on a warmed sample at an external laboratory (IDEXX Laboratories, Wetherby, UK) confirmed the marked hypoalbuminemia and hyperglobulinemia with a monoclonal band present in the gamma fraction (monoclonal gammopathy, Figure 5). The same analysis was repeated on a room temperature sample, with comparable results. Urinalysis revealed proteinuria with urine protein–creatinine (UPC) ratio of 1.44 (normal: <0.2) with adequate specific gravity at 1.036, and an inactive sediment. Urine electrophoresis (Laboklin, Germany) revealed severe unselective glomerular and tubular protein loss but no evidence for Bence Jones proteinuria. The patient tested negative for *Leishmania* antibodies (ELISA, IDEXX) as well as *Dirofilaria immitis* antigen, *Ehrlichia canis* and *Ehrlichia ewingii* antibodies, *Anaplasma phagocytophilum* and *Anaplasma platys* antibodies, and *Borrelia burgdorferi* antibodies (IDEXX SNAP 4Dx plus test).

Given all the findings, the patient was diagnosed with multiple myeloma with associated cryoglobulinemia. The patient was internally referred to the oncology department for consultation. A full chemotherapy protocol was proposed, consisting of melphalan and prednisolone. However, the owners elected palliative care and the patient was discharged from the hospital to continue a treatment protocol at home with prednisolone (prednisolone 0.5 mg/kg PO SID), after a wash out period of 72 h since the last dose of non‐steroidal anti‐inflammatory medication, and gabapentin (10 mg/kg PO BID). Approximately 5 weeks after the diagnosis, the patient deteriorated rapidly with worsening of the epistaxis, episodes of regurgitation and restlessness. Follow‐up haematology and biochemistry performed by the primary care veterinarian revealed progression of her anaemia (HCT: 19.8%; RI: 37.3%–61.7%) and azotaemia (urea: 44.7, RI: 2.5–9.6 mmol/L; creatinine: 706, RI: 44–159 µmol/L). Due to the poor prognosis and her quality of life, the patient was humanely euthanized. No necropsy was performed.

## 2 Discussion

Cryoglobulinemia is a rare phenomenon in both humans and animals (Kolopp‐Sarda and Miossec 2022; Kreutzfeldt and Browne 2023). Cryoglobulins are immunoglobulins that undergo reversible precipitation when exposed to low temperatures and re‐dissolve upon sample warming. The exact mechanisms of this process are not fully understood, although the solubility of the protein appears to be dependent on several factors apart from temperature, such as the pH, and the ionic strength of the solution (Gulli et al. 2017).

A classification scheme in human medicine separates cryoglobulins into three types. Type I consists of isolated monoclonal immunoglobulins, Type II consists of mixed cryoglobulins with a monoclonal component, and Type III consists of mixed/polyclonal cryoglobulins. This classification has also been adapted in veterinary literature (Hurvitz et al. 1977; Hickford et al. 2000). Type I cryoglobulins are associated with lymphoproliferative diseases, such as multiple myeloma, Waldenstrom macroglobulinemia and lymphoma (Napodano et al. 2021). In the veterinary literature, monoclonal cryoglobulinemia has been described in dogs (Braund et al. 1979; Stickle and Henkel 1995) and a cat (Hickford et al. 2000) with multiple myeloma, a dog with Waldenstrom's macroglobulinemia (Hurvitz et al. 1977) and two horses with lymphoma (Kreutzfeldt and Browne 2023; Traub‐Dargatz et al. 1985). Types II and III, also referred to as mixed cryoglobulins, have been reported in various veterinary species, including several horses and more recently in a rhesus macaque with glomerulonephritis (Sabnis, Gunson, and Antonovych 1984; Maede et al. 1991; Tang et al. 2017). The underlying cause of cryoglobulinemia in these cases has not been thoroughly investigated, although infectious or immune‐mediated causes have been suspected in some cases. In human medicine, infection with Hepatitis C virus is a well‐described condition often associated with cryoglobulinemia (Trejo et al. 2001; Gulli et al. 2017). The detection of cryoglobulins requires the observation of a cryoprecipitate formed in the serum, which re‐dissolves when warmed. Precipitation occurs more rapidly in cases of Type I cryoglobulinemia and is reportedly within 24 h (likely due to the increased concentration). However, the precipitation process is slower and may take several days in mixed/polyclonal cryoglobulins. The diagnostic process of detection of cryoglobulinemia in humans involves specific pre‐analytical and analytical guidelines, including sample collection in pre‐warmed syringes and tubes, and transport to the laboratory while the sample remains at 37°C. The sample should be allowed to clot at 37°C, and after separation of the serum, an aliquot should be stored at 4°C for approximately 7 days, for visual observation of the cryoprecipitate (Gulli et al. 2017; Kolopp‐Sarda and Miossec 2022). The precipitate can have variable forms, depending on the composition of the immunoglobulin (white, translucent, flocculent, gelatinous or crystalline). Strict guidelines are aimed at eliminating false‐negative results (Gulli et al. 2017; Kolopp‐Sarda and Miossec 2022; Motyckova and Murali 2011; Napodano et al. 2021), as cryoglobulins could precipitate in the clot at lower temperatures and remain undetected during the analysis. False positive results are rare and can occur by precipitation of lipids in lipemic samples in low temperatures, or observation of a cryofibrinogen precipitate in tubes when anticoagulants are used instead of serum (Kolopp‐Sarda and Miossec 2022). A similar phenomenon can be observed in human patients receiving anticoagulant therapy, which can result in the formation of fibronectin–heparin complexes and false‐positive cryoprecipitation (Sargur, White, and Egner 2010). In humans, quantification of the cryoglobulins can be performed with different specialized methods, after isolation of the cryoprecipitate (Gulli et al. 2017). Further characterization of the cryoglobulin precipitate can also be pursued (e.g., using immunofixation electrophoresis).

In this case, the described human recommendations were not followed, as the diagnosis of cryoglobulin was incidental and no further samples from this patient were available for analysis within the laboratory. Thus, some of the protein may have been lost during the sample clotting, and the true concentration could be higher than measured in the warmed sample. Total protein, albumin and globulin measurements were repeated in the leftover serum, with the marked discrepancy noted, as similarly reported in other cases in literature (Stickle and Henkel 1995). The marked differences seen in the total protein and globulin concentrations between the room temperature and warmed samples were thought to be due to the precipitation of cryoglobulin in the room temperature sample. Other reported artefacts of cryoglobulin precipitation notably include pseudoleukocytosis and pseudo‐thrombocytosis on machine counts, the presence of leukocyte inclusions (thought to represent cryoglobulin droplets) and extracellular deposits/clumps of smooth amorphous material in blood smears, and ‘loss’ of the monoclonal component in SPE (Ileri et al. 1998; Dalal and Brigden 2002; Sargur, White, and Egner 2010; Maitra et al. 2000). The presence of abundant deposits of amorphous material in the blood smear from this patient prompted further analysis and observation of the cryoprecipitate in this case. No falsely elevated cell counts were noted, as cell density on the smear appeared comparable to the automated results. A monoclonal component was clearly evident in both the room temperature and warmed samples; however, the possibility that the concentration of the monoclonal protein may have been underestimated cannot be ruled out, because the routine sample handling before serum separation may have resulted in partial ‘loss’ of the cryoglobulin.

Multiple myeloma is an uncommon malignant tumour in dogs (less common in other domestic species), accounting for approximately 8% of all haematopoietic malignancies in this species (Borgatti 2022). Diagnosis of multiple myeloma in dogs has traditionally relied upon the documentation of two out of the following four criteria: (1) bone marrow plasmacytosis (>20% of the bone marrow population), (2) osteolytic bone lesions (3), monoclonal gammopathy in SPE and (4) Bence Jones (light chain M‐protein) proteinuria (Borgatti 2022). Recently, the diagnostic criteria in human medicine were updated, in order to increase the ability to diagnose non‐secretory myelomas which, as the name suggests, are not characterized by a secretory product and thus an M‐component is not detected in the serum or urine (Brown et al. 2021). This case demonstrated a secretory component, evident by the detection of monoclonal gammopathy and, with the demonstration of neoplastic plasma cells in the bone marrow and spleen, definitive diagnosis followed the traditional criteria. The patient additionally exhibited myeloma‐related events, given the anaemia and renal disease. No osteolytic lesions were identified in the head and thorax CT, and no pain was elicited in external palpation/manipulation of the patient; however, a full body CT or necropsy was not performed. The prognosis of dogs with multiple myeloma is variable depending on response to chemotherapy (which was not pursued in this case), with some patients achieving one or multiple years of good quality of life, whereas others may respond only for a few months. The majority of patients, however, will not achieve complete remission (Vail 2020).

Prior to referral, the patient's presenting signs (epistaxis, nasal planum crusting and resolving skin damage on the lip) were originally thought to be a result of a more localized inflammatory disease, and symptomatic treatment with antibiotic and anti‐inflammatory medications was attempted with no response. Subsequent clinical (ulcerations in multiple mucocutaneous areas) and clinical pathology findings (precipitate in the blood smear and serum which disappeared after warming) allowed the recognition of cryoglobulinemia. Precipitation of cryoglobulins at extremities within vessels leads to vasculitis and/or vessel occlusion, with subsequent ischaemic necrosis. As the patient presented during winter, it is presumed that the lower temperature at the extremities and mucosal surfaces caused by cold exposure exacerbated this phenomenon. Clinical manifestations of cryoglobulin precipitation most commonly involve the skin (mucocutaneous ulcerations noted in this case), with renal involvement more rarely seen (Khwaja et al. 2022). As this phenomenon has been described in cases of lymphoproliferative disorders, and given the presence of pancytopenia, a systemic cause (likely neoplastic) was suspected in this case. The subsequent bone marrow and spleen cytology, and the monoclonal gammopathy on SPE, confirmed the final diagnosis of multiple myeloma, as an underlying cause of the cryoglobulinemia. The presence of epistaxis in this patient was attributed to hyperviscosity syndrome, associated with the presence of M‐component. It is characterized by increased serum viscosity, causing sluggish blood flow and tissue hypoperfusion. Hyperviscosity syndrome is a well‐described consequence of multiple myeloma, occurring in approximately 20% of dogs (Borgatti 2022), characterized by altered blood flow properties, often causing bleeding, particularly from mucosal surfaces (Yu et al. 2020). Other clinical manifestations of hyperviscosity can also include neurological signs (e.g., seizures and depression), ophthalmic abnormalities (e.g., retinal haemorrhages and detachment) and renal failure. No neurological or ophthalmic abnormalities were observed in this case. Both hyperviscosity and cryoglobulinemia are mentioned in the literature as potential underlying mechanisms in the pathogenesis of renal disease (Gulli et al. 2017; Borgatti 2022; Khwaja et al. 2024) and likely contributed in this case, although multiple concurrent mechanisms such as renal infiltration by neoplastic plasma cells, or renal amyloidosis could also be occurring.

Extremely rarely, cryoglobulins can act as cold agglutinins, resulting in immune‐mediated haemolysis (Pujol et al. 1998; Khwaja et al. 2024). This was not seen in this case. It should be noted that the majority of cryoglobulinemia cases are not associated with cold agglutinin disease and, despite the similar association with cold temperature, these two entities should not be confused. Cold agglutinins are cold‐reactive antibodies that are able to agglutinate erythrocytes and may cause haemolysis, as well as possible cold‐induced ischaemic manifestations in peripheral skin sites (e.g., ears, toes, tip of the nose).

This case describes the presence of cryoglobulin, an extremely rare finding, in the peripheral blood of a dog, associated with multiple myeloma. This report raises awareness about the possible interference of cryoglobulin on protein measurement and the importance of a simple and cost‐effective blood smear examination. Recognition of this phenomenon allowed a more accurate protein measurement, unmasking the degree of hyperglobulinaemia and steering the diagnosis towards a lymphoproliferative disorder. Additional testing, in particular serum immunofixation electrophoresis, could have been valuable to further characterize the monoclonal gammopathy, by determining the exact immunoglobulin class involved. Although Bence Jones proteinuria was not visible in standard urine electrophoresis, its presence cannot be ruled out as urine immunofixation electrophoresis was not performed to further investigate for the presence of free light chains.

## Author Contributions

**Myrto Spyropoulou**: writing–original draft. **Ivan Montanes‐Sancho**: writing–review and editing. **Adam G. Gow**: writing–review and editing. **Suzanne Bussey**: supervision, writing–review and editing.

## Ethics Statement

The authors have nothing to report.

## Conflicts of Interest

The authors declare no conflicts of interest.

### Peer Review

The peer review history for this article is available at https://publons.com/publon/10.1002/vms3.70084.

## Data Availability

Data sharing is not applicable to this article as no new data were created or analysed.
